# Assessment of Safety, Tolerability, Pharmacokinetics, and Volume-Dependent Conductive Hearing Loss in Healthy Volunteers: First-in-Human, Open-Label, Placebo-Controlled Study of a Single Intratympanic Injection of AC102

**DOI:** 10.1097/MAO.0000000000004568

**Published:** 2025-08-13

**Authors:** Cris Lanting, Sybren Robijn, Michael Nieratschker, Christin Galetzka, Alena Meis, Hans Rommelspacher, Sandra Ahoud-Schoenmakers, Edith Lackner, Markus Zeitlinger, Martin Bauer, Christoph Arnoldner, Ronald Pennings, Reimar Schlingensiepen

**Affiliations:** ∗ Department of Otorhinolaryngology, Radboudumc; † Donders Institute for Brain, Cognition and Behaviour, Nijmegen, The Netherlands; ‡ Department of Otorhinolaryngology, Head and Neck Surgery, Medical University of Vienna, Vienna, Austria; § AudioCure Pharma GmbH, Berlin, Germany; ∥ Radboud Technology Center Clinical Studies, Nijmegen, The Netherlands; ¶ Department of Clinical Pharmacology, Medical University of Vienna, Vienna, Austria

**Keywords:** AC102, Conductive hearing loss, Inner ear therapy, Intratympanic injection, Phase 1 trial, Safety

## Abstract

**Objective:**

The objective of this study was to assess the safety and tolerability of the intratympanic delivery of AC102, a novel pharmaceutical therapy based on a thermosensitive gel for preventing and treating a range of hearing impairments, including sudden sensorineural hearing loss. We studied this in healthy, normal-hearing volunteers to evaluate any change in hearing thresholds.

**Study Design:**

An open-label, placebo-controlled, ascending single-dose, multicenter phase 1 clinical trial.

**Setting:**

The study was conducted in two centers (blinded for reviewing purposes).

**Subjects:**

Forty-two normal-hearing healthy volunteers younger than 40 years of age were eligible for enrollment in the study.

**Intervention:**

A single intratympanic injection of a thermosensitive gel, either a placebo or containing AC102, to the middle ear.

**Main Outcome Measure:**

The primary objective of this study was to assess the safety and tolerability of a single intratympanic injection of ascending volume of placebo and ascending volume and concentration of AC102. The secondary objective was to determine single-dose pharmacokinetics of intratympanically injected AC102 and to evaluate any change in hearing thresholds in healthy male and female subjects.

**Results:**

The intratympanic delivery of AC102 thermogel was safe and well tolerated in healthy volunteers with normal hearing, with no permanent adverse events recorded. A mild and temporary volume-dependent conductive hearing loss in the higher frequencies was observed irrespective of AC102 or placebo, which did not result in long-term changes in hearing. Other transient adverse events related to the injection procedure were largely similar between placebo gel and AC102 suspension, consisting mainly of mild ear discomfort, tinnitus, otalgia, and the formation of a droplet of blood at the injection site.

**Conclusions:**

The results of this phase 1 clinical trial suggest that the intratympanic delivery of AC102 is a safe and well-tolerated approach for drug delivery to the inner ear in healthy volunteers with normal hearing. As temporary, volume-dependent, conductive hearing losses were observed in the higher frequencies, it is recommended that patients are counseled on a short-term increase in hearing thresholds following injection for conditions such as sudden hearing loss.

## INTRODUCTION

Despite numerous efforts, to date, no approved pharmacotherapies are available for the treatment of sudden sensorineural hearing loss (SSNHL). The treatment of hearing loss with systemic or intratympanic glucocorticoid steroids, which have anti-inflammatory and immunosuppressive properties, is recommended in many countries and is regarded as the standard of care. However, there is a lack of clear evidence for the significant beneficial effects of glucocorticoids in treating SSNHL (1). In addition, such treatment may not be justified due to the possible side effects, including hypertension, behavioral disturbances, and diabetes mellitus. Although the root cause of SSNHL is often unknown (2–4), it likely affects the hair cells and spiral ganglion neurons (SGNs) of the auditory nerve. Importantly, neither the hair cells nor the SGNs can be replaced once lost. Therefore, it is crucial to have an immediate and target-specific intervention to prevent any further damage to these cells and to prevent acute hearing loss from becoming permanent.

Getting drugs or medication into the inner ear can be a challenging task due to the presence of the blood-labyrinth barrier that protects the inner ear and spiral ganglion neurons (SGNs). Therefore, a systemically administered medication is less likely to reach these target tissues at levels sufficient to have a therapeutic effect without the risk of systemic adverse reactions (5). Different approaches have been explored to target the inner ear effectively, including local delivery via intratympanic injection, systemic administration combined with permeability enhancers, and direct infusion or injection into the cochlea (6–9). Among these, local delivery via intratympanic injection of drugs formulated in a thermosensitive gel has emerged as a promising strategy for prolonged drug delivery to the inner ear (10).

The thermosensitive gel can be designed to be fluid at room temperature and thicken at body temperature, thus remaining in the middle ear, allowing for slow and continuous drug release (10). Once administered, the drug can diffuse through the round window membrane (RWM) and surrounding mucosa into the inner ear, where it can reach the target cells and exert its therapeutic effects (11).

AC102 is a novel pharmaceutical therapy for preventing and treating a range of hearing impairments, including acute hearing loss, tinnitus, and trauma due to cochlear implant electrode insertion; all these conditions have a high unmet medical need (12,13). In an animal model of hearing loss, AC102 prevented noise-triggered loss of outer hair cells (OHCs) and reduced inner hair cell (IHC) synaptopathy, suggesting the role of AC102 in reconnecting auditory neurons to their sensory target cells. In vitro experiments of a neuronal damage model revealed that AC102 protected cells from apoptosis and promoted neurite growth (14). Moreover, AC102 significantly preserves hearing thresholds across the whole cochlea. It confines the cochlear trauma to the directly mechanically injured area following cochlear implantation and associated electrode insertion trauma. It significantly preserves auditory nerve fibers and inner HC synapses throughout the cochlea (15).

This paper presents the results of a phase 1 clinical trial evaluating an intratympanic injection's safety, tolerability, and pharmacokinetics with either AC102 suspension or placebo gel in normal-hearing healthy volunteers. Furthermore, we discuss the impact of the gel on pure tone audiometry thresholds, which is essential since a temporary increase in thresholds may be anticipated following injection for conditions like sudden hearing loss (16).

## MATERIALS AND METHODS

### Study Design

**FIG. 1:**
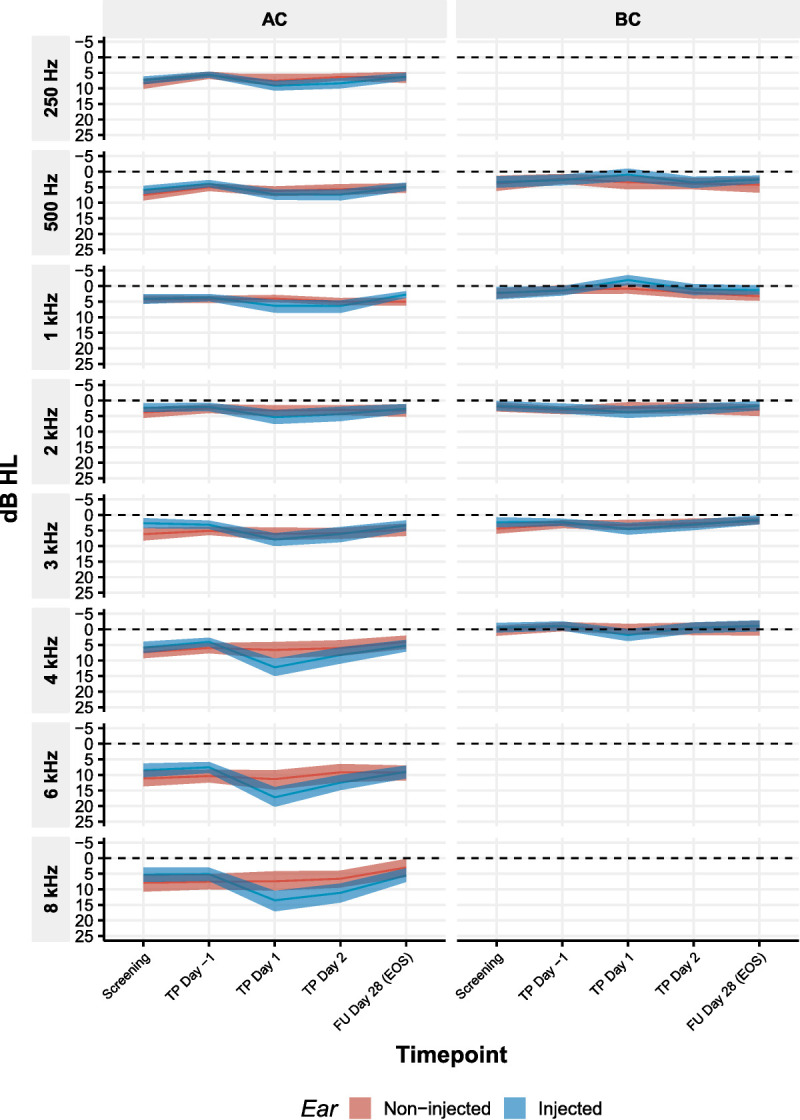
The average pure tone average (PTA) thresholds (n = 42 subjects) for air conduction (left column, AC) and bone conduction (right column) during screening, transtympanic injection day −1 (TP day −1), directly after the transtympanic injection (TP day 1), the day after (TP day 2), and at follow-up day 28 at the end of the study (FU day 28 EoS). The frequency tested, ranging from 0.25 to 8.0 kHz, is displayed in each panel and represents the threshold values for the injected ear (blue) and the noninjected ear (red). The data points depict the mean threshold, with the 95% confidence interval around the mean threshold determined using bootstrapping. The dashed line signifies the 0-dB line.

This phase 1 study was an open-label, placebo-controlled, ascending single-dose study in healthy, normal-hearing male and female subjects. Forty-two subjects were enrolled and treated. The study was conducted at the Radboud University Medical Center, Nijmegen, and the Medical University of Vienna. Patients were recruited through Link2Trials (https://www.link2trials.com/clinical-trials/). Compensation was provided for the subjects' time, travel, inconvenience, and discomfort as a result of participation in the study. The Medical Ethics Review Committee reviewed and approved the study (CCMO NL72516.056.20; EUCTR2019-004969-40-NL). The primary objective of this study was to assess the safety and tolerability of single intratympanic injections of ascending volumes of placebo and ascending volumes and concentrations of AC102. The secondary objective was to determine single-dose pharmacokinetics of intratympanically injected AC102 in healthy male and female subjects. The related endpoints are described in Table 1. In addition, pure tone audiometry (PTA) and objective measures of hearing, such as otoacoustic emissions (OAEs) and brainstem evoked response audiometry (BERA), were recorded in both the injected and noninjected ear (Fig. 1).

**TABLE 1:** Primary and secondary objectives and endpoints

Objectives	Endpoints
*Primary*	
To assess the safety and tolerability of single ascending volumes of placebo and ascending volumes and concentrations of AC102 by an intratympanic injection in healthy male and female subjects	• Treatment-emergent (serious) adverse events (TE[S]AEs)
	• Adverse events of special interest (AESIs):
	○ Permanent sensorineural hearing loss
	○ Persistent conductive hearing loss
	○ Persistent tinnitus
	○ Persistent vestibular vertigo
	○ Persistent tympanic membrane perforation
	○ Infections of the outer, middle, and inner ear
	• Concomitant medications
	• Clinical laboratory tests:
	○ Hematology
	○ Chemistry
	○ Urinalysis
	• Vital signs:
	○ Pulse rate (beats per minute [bpm])
	○ Systolic and diastolic blood pressure (mm Hg)
	○ Body temperature
	• Electrocardiogram (ECG):
	○ Heart rate (HR) (bpm)
	○ QTcF (corrected QT interval calculated using Fridericia's method)
	• Otological assessments:
	○ Otoscopy
	○ Tympanometry
	• Audiological assessments:
	○ Pure tone audiometry (PTA)
	○ Otoacoustic emission (OAE)
	○ Brainstem evoked response audiometry (BERA)
	• Assessments of the vestibular system:
	○ Video head impulse test (vHIT)
	○ Frenzel goggles
	○ Vestibular-evoked myogenic potential (VEMP) recording
*Secondary*	
To determine single-dose pharmacokinetics (PK) in plasma of intratympanically injected AC102 in healthy male and female subjects	• The area under the plasma concentration-time curve from zero to infinity (AUC_0-inf_)
	• The maximum plasma concentration (*C*_max_)
	• The area under the plasma concentration-time curve from zero to time of the last measured concentration above the limit of quantification (_AUC0-tlast_)
	• The time to reach maximum plasma concentration (*t*_max_)
	• The apparent terminal disposition rate constant (λ_z_) with the respective half-life (*t*_½_)
	• Other parameters, including apparent volume of distribution (Vz/F), apparent total body clearance (CL/F), and other parameters as appropriate and possible, as well as dose-adjusted parameters

**FIG. 2:**
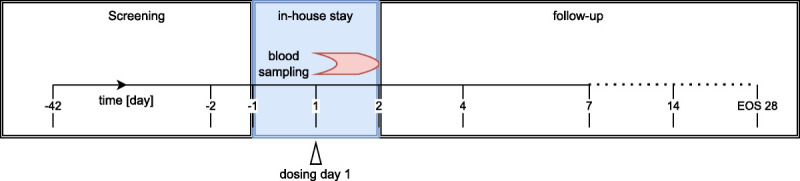
Study design. After screening, eligible subjects were invited to the treatment period/in-house stay. After reconfirming eligibility on day −1, the subjects were dosed on day 1 and monitored at the study site for at least 24 hours. Outpatient visits were performed on day 4 after administration of the study medication and on day 7 (±1 d) and day 14 (±2 d) if any adverse events (AEs) were observed during the visit of day 4. The end-of-study (EoS) visit occurred on day 28 (±4 d).

After screening, eligible subjects were invited to the treatment period/in-house stay. After reconfirming eligibility on day −1, subjects were dosed on day 1 and monitored at the study site for at least 24 hours before discharge. The first follow-up was performed as an outpatient visit on day 4. If any adverse events (AEs) were observed during the visit on day 4, outpatient visits were conducted on day 7 (±1 d) and day 14 (±2 d). Otherwise, the well-being of the subjects was checked during a phone call on day 7 and day 14. The end-of-study (EoS) visit occurred on day 28 (±4 d) (Fig. 2).

### Study Population

Healthy adult subjects not older than 40 years of age were included in the study to reduce the risk of presbycusis as a confounding factor. As systemic effects or toxicities after local administration of AC102 are considered minimal, female subjects practicing double-barrier contraception could participate. After informed consent, subjects were screened for eligibility. An extensive list of inclusion and exclusion criteria was used, and subjects had to fulfill these criteria to participate in the study (see Supplemental Table 1, http://links.lww.com/MAO/C130).

### Study Assessments

Safety and tolerability assessments, including AE recording, concomitant medications review, clinical laboratory tests, vital signs recording, 12-lead ECG testing, physical examinations, otological assessments, audiological assessments, and assessments of the vestibular system, were performed up to the EoS. Audiological measurements included tympanometry and pure tone audiometry. The function of the outer hair cells (OHCs) was assessed by the presence or absence of distortion-product otoacoustic emissions (DPOAEs) and transient-evoked otoacoustic emissions (TEOAEs). In addition, brainstem evoked response audiometry (BERA) was performed to obtain objective hearing measurements. Vestibular function was assessed by Frenzel goggle evaluation, ocular and cervical vestibular evoked myogenic potential (o- and cVEMP), and by video head impulse test (vHIT) of all semicircular canals. All audiovestibular evaluations were performed according to current clinical standards. Blood samples for the PK analysis of AC102 were collected up to 24 hours post-AC102 administration. The timing of assessments is shown in Supplemental Table 2, http://links.lww.com/MAO/C131.

Adverse events (AEs), treatment-emergent adverse events (TEAEs), and adverse events of special interest (AESIs) were noted on each study visit (e.g., permanent sensorineural hearing loss, persistent conductive hearing loss, persistent tinnitus, persistent vestibular vertigo, persistent tympanic membrane perforation, and infections of the outer, middle, and inner ear). Causality or degree of relatedness with treatment was assessed by assessing the temporal association with the treatment. Fisher's exact test was used to determine if there was a significant association between the number of TEAEs and the group (placebo or AC102). Since sex differences in pharmacokinetics predict adverse reactions in women (17), we analyzed the total number of AEs as a function of sex and treatment group, as well as their interaction.

### Study Cohorts

Sixteen single-dose cohorts (n = 3) were dosed in this study according to the traditional 3 + 3 design for ascending dose studies (18). The injections were performed by two surgeons, one per site (R.P. and C.A.), and participants were assigned to the cohorts based on availability. Each cohort received a placebo or AC102 suspension as an intratympanic injection in ascending order by volume or concentration (Table 2). AC102 is formulated as a suspension in a thermosensitive vehicle. The basis of the thermosensitive formulation used within this study as a placebo is a poloxamer. The vehicle gel was tested for each increased volume before the AC102 suspension was administered in the following cohort. For the first (C1) and the fifth (C5) single-dose cohort, 50-μl placebo or AC102, respectively, were administered for the first time in humans. Therefore, sentinel dosing of one subject occurred at least 2 weeks before dosing the remaining two subjects as recommended (19). This allowed careful monitoring of the first signs of AEs. Subjects in the first cohorts were planned to be dosed with a fixed volume, as outlined in Table 2. For the following cohorts (C10–C12), the aim was to fill the middle ear space of the individual subject with the volume injected based on the size and anatomy of the middle ear. Subjects were dosed until backflow or other AES/inconveniences occurred, up to a maximum volume of 800 μl.

**TABLE 2:** Dosing in treatment cohorts

Cohort	Number of Subjects—AC102: Placebo	F/M	Volume	Concentration AC102	Mean Age (SD)
C1a*^a^* C1b	0:1 0:2	2/1	50 μl	0 mg/ml	26.7 (7)
C2	0:3	1/3	100 μl	0 mg/ml	22.3 (2.1)
C3	0:3	1/3	200 μl	0 mg/ml	22.0 (4.0)
C4	0:3	3/3	400 μl	0 mg/ml	26.0 (3.6)
C4R*^b^*	0:3		400 μl	0 mg/ml	
C11*^b^*	0:3	5/6	up to 800 μl	0 mg/ml	27.0 (5.6)
C11R*^b^*	0:3		up to 800 μl	0 mg/ml	
C5a*^a^* C5b	1:0 2:0	1/3	50 μl	6 mg/ml	33.0 (8.2)
C6	3:0	1/3	100 μl	6 mg/ml	22.3 (1.2)
C7	3:0	1/3	100 μl	12 mg/ml	28.3 (9.2)
C8	3:0	2/3	200 μl	12 mg/ml	21.7 (2.1)
C9*^b^*	3:0	3/0	400 μl	12 mg/ml	26.3 (11.9)
C12*^b^*	3:0	2/3	up to 800 μl	12 mg/ml	30.3 (4.6)
C10*^b^*	3:0	3/0	Maximum feasible dose	12 mg/ml	25.0 (1.0)

*^a^*In cohorts C1a and C5a, one subject received sentinel dosing at least 2 weeks before the remaining two subjects (C1b, C5b).

*^b^*Dosing in C4 was repeated (C4R) to include a pressure-equalizing puncture. In C11, dosing was repeated (C11R) at a second center to assess differences in application techniques (e.g., needle form). The final cohort (C10, C12) involved two centers to confirm the maximum feasible dose based on prior cohorts.

Administration of higher volumes from >200 μl occasionally required a second puncture of the tympanic membrane to release air from the middle ear. Each prescheduled cohort could be conducted at the volume and dose outlined in the protocol or adjusted to the maximum injectable volume (in the cohorts with larger volumes).

Dosing in C4 was repeated (C4R) to adapt the administration procedure by including an additional pressure-equalizing puncture. Dosing in C11 was repeated (C11R) in the second study center to evaluate differences in the application techniques (e.g., needle form) the investigators were using. The last cohort was also performed in two centers (C10, C12). It was used to repeat and confirm the maximum feasible dose selected based on evaluating all previous cohorts (safety/tolerability and practicability of administration).

### Study Medication and Application

AC102, a small lipophilic molecule, 6-fluoro-9-methyl-pyridoindole, is formulated as a suspension in a biocompatible vehicle gel. The formulation is resuspended prior to injection through gentle shaking and applied through a lumbar puncture needle (Whitacre or Quincke-type spinal needles, 25G, 90-mm length) by intratympanic injection into the tympanic cavity.

For the application procedure, the head was placed in a tilted position, approximately 45° toward the nontreated contralateral ear, with the ear to be treated pointing upward. After cleaning, local anesthetic was applied topically onto the tympanic membrane (Lidocaine 10% pump spray) and left in place for approximately 30 minutes. After this, it was carefully suctioned off. The administration of study medication was performed preferably in the posterior-inferior quadrant of the tympanic membrane (Q3). The tympanic membrane was punctured with a 25G lumbar puncture for injection, and the content of the syringe was applied carefully into the middle ear while not touching the mucosa or the RWM. For higher volumes, the injection was stopped based on observed backflow or increased pressure during injecting. The application was performed slowly so the gel could spread evenly within the middle ear (10). The amount of injected volume of the compound into the middle ear was determined based on the injected volume, the volume left in the syringe, and the estimated amount of volume that flowed back based on the suctioned volume.

Following the study medication administration, subjects remained in the same position for 30 minutes so that the medication could reside in the round window niche, allowing for diffusion of the active substance across the RWM. Subjects were asked to refrain, if possible, from head movements, swallowing, yawning, sneezing, or talking during the resting period.

### Data Analysis

To assess the safety of the procedure, we assessed changes in bone and air conduction thresholds, the BERA thresholds, and the TEOAE and DPOAE signal-to-noise ratio. Using linear mixed models (defined in R using LME4), we first defined a model with the air and bone conduction threshold (dB HL). We tested for changes over time after injection, defining the subject Id as a random effect for individual variability between subjects. A similar model was used to assess BERA thresholds and OAEs between the two time points (day −1 vs day 28, EoS), where subject Id was defined as a random effect for individual variability between subjects and included a factor coding the injected versus contralateral ear.

To visualize the threshold changes over time, we defined three volume regimes. All data from the placebo and AC102 gel with injected volume <100 μl were defined as a small volume, between 100 and 340 μl as a medium volume, while >340 μl was described as a large volume. Finally, we analyzed the air conduction threshold change as a function of volume injected for each frequency to assess frequency-dependent effects.

## RESULTS

### Enrollment and Evaluation of Intratympanic Injections

Forty-two subjects were enrolled and underwent intratympanic injection with the placebo or AC102 gel. Overall, the mean age was 26.0 years (18 to 40 years), and the mean BMI was 23.71 kg/m^2^ (18.6 to 31.3 kg/m^2^). Twenty-three of the 42 enrolled subjects (54.8%) were female, and 19 of the 42 enrolled subjects (45.2%) were male. Demographic and baseline characteristics were generally balanced among the cohorts. Placebo overall contained more male subjects, and AC102 contained more female subjects.

During the administration of the study medication in the first C4 cohort, backflow of gel to the external auditory canal was observed mainly due to pressure building up in the middle ear during the injection of gel volumes >200 μl. Therefore, the procedure for the intratympanic administration of study medication was adjusted by the recommendation of using a second pressure-equalization technique, as previously described (20,21). Dosing in C4 was repeated (C4R) to implement the new administration procedure, which included a second pressure-equalizing puncture of the tympanic membrane. Dosing in C4 was repeated (C4R) before the same volume of AC102 suspension was applied in C9. One subject in the C4R cohort received only 15 μl of the assigned 400-μl placebo because the procedure had to be abandoned due to anxiety and stress experienced by the subject.

Dosing in C11 was repeated (C11R) in the other study center to evaluate potential differences in application techniques. Investigators used two types of needles (Whitacre or Quincke-type spinal needles, 25G, 90-mm length). The last cohort (C10) was used to replicate (C12) and confirm the maximum feasible dose selected based on evaluating all previous cohorts (safety/tolerability and practicability of administration). All 21 subjects who received a single dose of AC102 (i.e., subjects in cohorts C5, C6, C7, C8, C9, C12, and C10) were included in the pharmacokinetic analysis. It was not necessary to repeat more cohorts to assess safety and tolerability.

### Adverse Events Assessments

There were no severe adverse events or treatment-emergent adverse events (TEAEs) leading to study discontinuation or adverse events of special interest (AESIs) in this study (Tables 3 and 4). All subjects experienced at least one TEAE; in total, 138 TEAEs were experienced by 21 subjects receiving a placebo, and 160 TEAEs were experienced by 21 subjects receiving AC102. Most TEAEs were assessed to be mild, and there were no severe TEAEs.

**TABLE 3:** Summary of adverse events (AEs)

TEAEs	Placebo	AC102	Total
By severity	Total (n = 21)	Total (n = 21)	
Mild	128	157	285
Moderate	10	3	13
Severe	0	0	0
By relationship			
Definitely	14	36	50
Probably	91	81	172
Possibly	10	25	35
Unlikely related	14	14	28
Not related	9	4	13
Any	138	160	298
SAE	0	0	
Deaths	0	0	
TEAE leading to study discontinuation	0	0	
AESIs	0	0	

AE indicates adverse event; SAE, serious adverse event; TEAE, treatment-emergent adverse event; AESI, adverse event of special interest.

**TABLE 4:** Treatment emergent adverse events reported by more than five subjects in either group

System Organ Class (Term)	Preferred Term	Number of Events	% of Total
Ear and labyrinth disorders	Ear discomfort	77	26%
	Tinnitus	23	8%
	Ear pain	21	7%
	Vertigo	18	6%
	Hypoacusis	14	5%
	Deafness unilateral	10	3%
	Acoustic stimulation tests abnormal	6	2%
General disorders and administration site conditions	Injection site hemorrhage	8	3%
Investigations	Acoustic stimulation tests abnormal*^a^*	9	3%
Nervous system disorders	Headache	15	5%
Respiratory, thoracic, and mediastinal disorders	Oropharyngeal pain	10	3%
	Throat irritation	6	2%
Total		217	73%

*^a^*Conductive hearing loss determined during PTA threshold testing (i.e., an air-bone gap between air conduction thresholds and bone conduction thresholds).

Most TEAEs were considered related to the study medication (i.e., either placebo or AC102) or the application procedure, as they had a clear temporal association with the study procedure. Of the subjects receiving placebo, 115 TEAEs were reported, and 142 TEAEs were experienced by subjects receiving AC102. TEAEs and AESIs that were reported by more than five subjects in the placebo group or by more than five subjects in the AC102 group included ear discomfort, tinnitus, ear pain, temporary vertigo, conductive hearing loss determined during PTA testing, diminished hearing (self-reported), oropharyngeal pain, and throat irritation (Table 4). In addition, we observed the formation of tiny droplets of blood at the injection site, turning into a hemorrhagic crust on the tympanic membrane.

Most of these commonly occurring related TEAEs were reported at a similar frequency in the placebo and AC102 groups. Supplemental Table 3, http://links.lww.com/MAO/C132, shows the number of AEs dependent on sex and treatment group. Note, however, that the groups are not balanced, making a straightforward comparison difficult. The placebo group consists of 8 female and 13 male subjects, and the AC102 group consists of 15 female and 6 male subjects. A generalized linear model using a Poisson distribution was used to assess the number of AEs per subject dependent on sex and treatment group. There was no statistically significant association between the number of TEAEs for the treatment group (*z* = −1.41, *p* = 0.16), nor the variable sex of the subjects (*z* = −1.17, *p* = 0.24). The TEAEs were most likely the result of the administration procedure and/or the mass effect of the gel.

Most TEAEs considered related recovered within 1 week after administration. There were three related events of ear discomfort (two subjects in C7 and one subject in C8) and one related event of subjectively diminished hearing (one subject in C8) that were not resolved by the end of the study (EoS). The subjects with ear discomfort were followed up after EoS, and all reported recovery within 4 weeks. The subject with subjectively diminished hearing showed hearing thresholds at EoS that were not different from day −1 at the frequencies tested, both for air and bone conduction. This subject reported recovery after 4 weeks after EoS.

In general, changes from baseline for all clinical laboratory evaluations, vital signs, and ECGs were small and within the range of clinical variation. No trends were observed. Otoscopic abnormalities caused by intratympanic administration of study medication either recovered or were considered no longer clinically significant by day 4. At the EoS, all subjects, except one in C8 (who still had a blood crust on the tympanic membrane), had a normal appearance of the treated ear. The tympanic membrane was closed (based on otoscopy and tympanometry) for all subjects within 4 days, independent of whether a secondary puncture hole was made to release air. Also, no myringosclerosis was seen at the EoS. No complications or negative influences on the healing process were observed, e.g., by the gel or AC102.

### Audiovestibular Evaluations

Bone conduction thresholds remained unchanged after administration of placebo or AC102, and no sensorineural hearing loss was observed throughout the trial. The function of the OHCs, as assessed by the presence or absence of DPOAEs and TEOAEs, did not change after intratympanic administration of study medication (measured on day 28, i.e., EoS, χ^2^ = 1.2518, *df* = 1, *p* = 0.26). There were no clinically relevant changes in inner ear and auditory nerve function (assessed by BERA). Results showed no change in the BERA threshold between day −1 and EoS (*p* = 0.3), nor between the injected or contralateral ear (*p* = 0.7) (Supplemental Fig. 1, http://links.lww.com/MAO/C133).

**FIG. 3:**
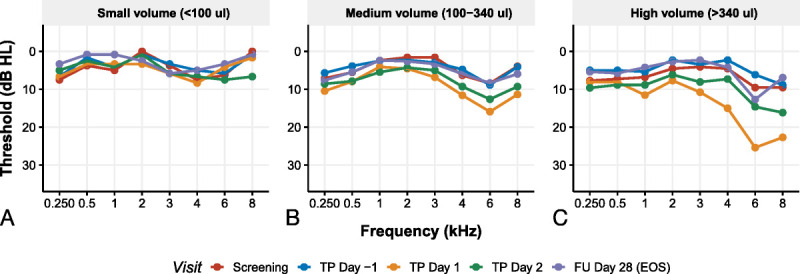
The average PTA air conduction threshold for different volume categories at various visits. The average threshold for screening, TP day −1, TP day 1, TP day 2, and follow-up (FU) day 28 at the end of study (EoS) are displayed in red, blue, orange, green, and purple, respectively. Panel *A* displays the average threshold for cases where a small volume (<100 μl) was injected, panel *B* shows the average threshold for cases where a medium volume (100–340 μl) was injected, and panel *C* presents the average threshold for cases where a high volume (>340 μl) was injected. The threshold is plotted against frequency.

To further assess the safety of the procedure, we assessed changes in bone and air conduction thresholds following pure tone audiometry. No significant change over time was observed in bone conduction thresholds (*F*[4,1743] = 1.05, *p* = 0.38). In air conduction thresholds, a significant effect of time was observed (*F*[4,2811] = 27,7, *p* < 0.001) when pooling data over placebo and the AC102 group. No significant differences in air conduction thresholds were observed between placebo and AC102 (*p* = 0.71, with a mean difference between placebo and AC102 of −0.47 dB; 95% confidence interval [−2.14, 1.20 dB]). This threshold increase depended on the volume injected (*F*[1,37.6] = 4.59, *p* = 0.03876). A noticeable effect was observed starting at volumes exceeding 100 μl (Fig. 3). A substantial increase in thresholds was noticed immediately postinjection, with the average increase in thresholds reaching 6.6, 15.9, and 11.4 dB at 4, 6, and 8 kHz, respectively, for medium volume injections, and 8.2, 23.6, and 20.9 dB at 4, 6, and 8 kHz for high volume injections (as depicted in Fig. 3, panels B and C, in orange). Despite some residual effect observed on day 2 postinjection, the thresholds eventually returned to baseline after 28 days. No effect of concentration of AC102 was observed on the air conduction thresholds.

**FIG. 4:**
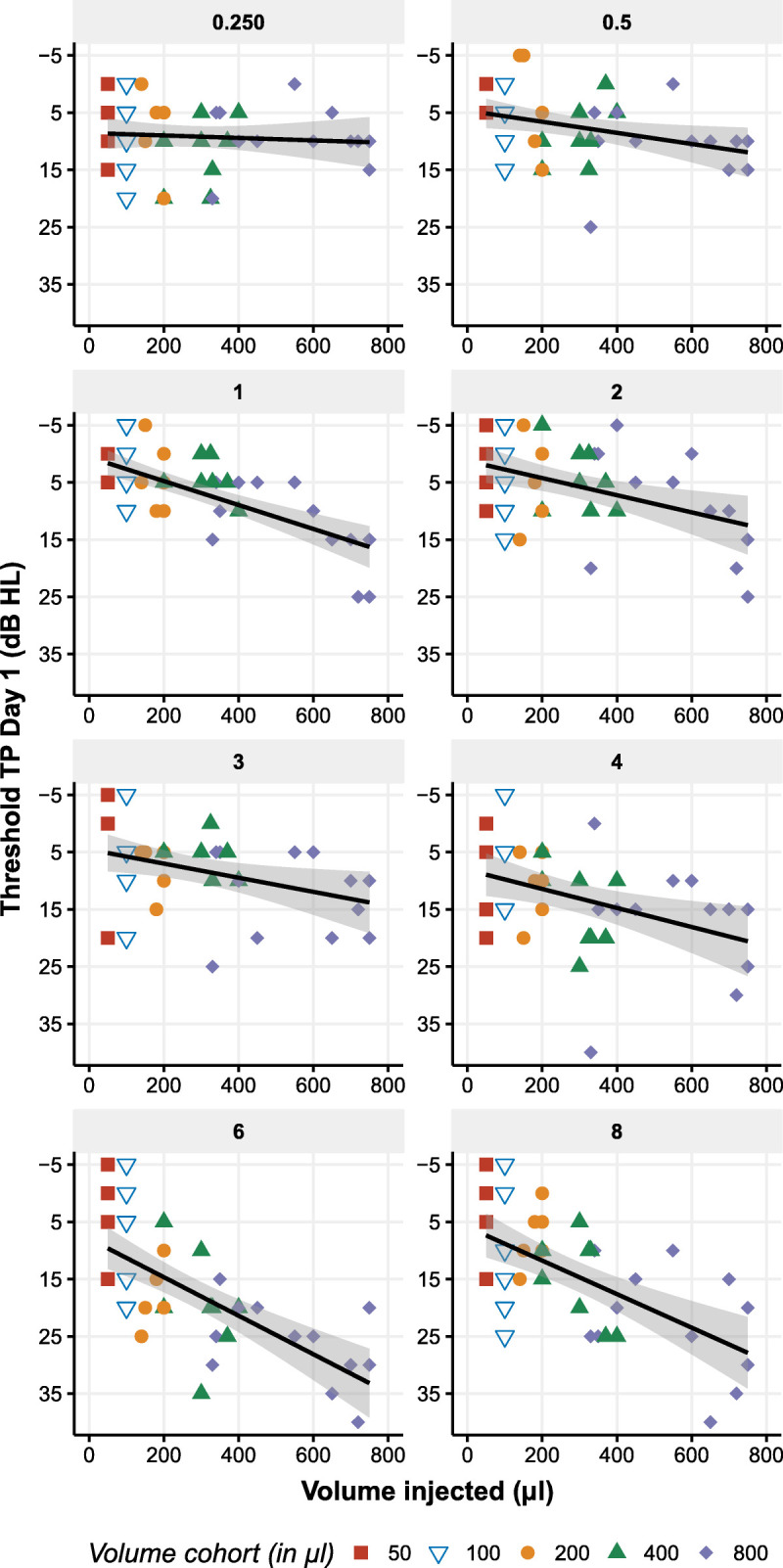
The average air conduction threshold versus the injected volume of either placebo or AC102 on the day of injection. Each panel showcases the frequency tested, ranging from 0.25 to 8.0 kHz, and displays the threshold measured after injection for the injected ear (threshold at TP day 1 in dB HL). The color code in each panel represents the volume cohorts, with red representing 50 μl, blue/white triangle representing 100 μl, orange circle representing 200 μl, green triangle representing 400 μl, and purple diamond representing 800 μl. The *x*-axis displays the exact volume injected for each participant, and the *y*-axis shows the effect of the injected volume on the threshold, with the most significant impact seen between 4 and 8 kHz. For each frequency, a linear fit with the confidence interval around the mean is presented (fit in black, confidence interval in gray).

An alternative approach to analyzing the results is plotting the air conduction thresholds against the injected volume (Fig. 4). Once again, the most pronounced effects were observed immediately after injection (threshold TP day 1), particularly at higher frequencies (i.e., 4–8 kHz), with a maximum threshold of 25 to 35 dB at 6 kHz for injections exceeding 500 μl. In comparison, the threshold at 1 kHz was much less affected, reaching a maximum of only 15 dB for injections over 500 μl. In summary, the intratympanic injection of a thermosensitive hydrogel causes a temporary air conduction threshold shift related to the fluid being injected into the middle ear.

### Pharmacokinetic Profile of AC102 After Injection

**FIG. 5:**
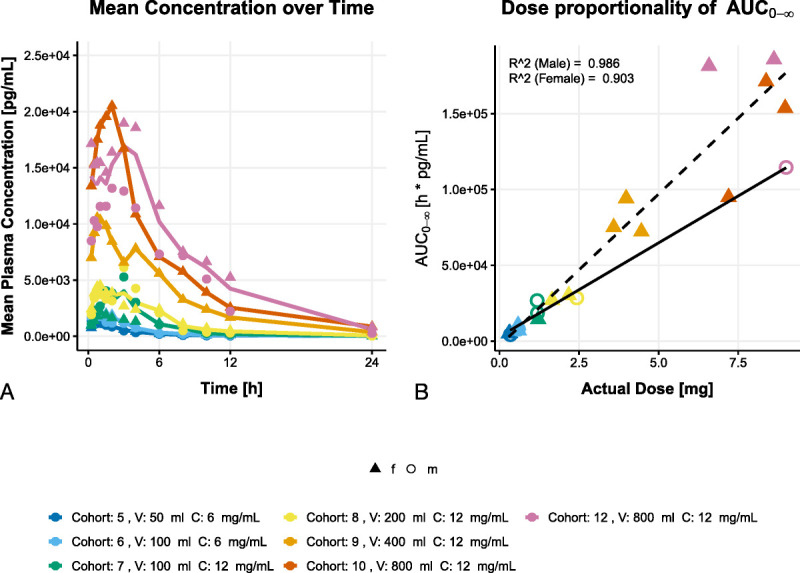
Pharmacokinetic profile of a single AC102 intratympanic injection in plasma up to 24 hours. Panel *A* shows the mean blood plasma concentration for each cohort over time (0–24 h postinjection). Panel *B* shows the area under the curve (AUC_0-∞_) as a function of the actual dose (mg) for each subject. The color code reflects the specific cohort, and the symbols reflect the sex. For both sexes, a separate regression line depicts the relation of the dose and the AUC_0-∞_.

Following a single intratympanic dose of AC102, maximum plasma concentrations were reached approximately 15 minutes to 4 hours after dosing. The median time to reach a maximum, *t*_max_, was 1 to 2 hours. Plasma concentrations gradually decreased after that (Fig. 5A). The geometric mean half-life (*t*_1/2_) ranged from 3.0 (in C5, sentinel dose) to 5.8 hours (in C10, maximum dose). For almost all subjects, plasma concentrations could still be measured 24 hours postdose, the last time point tested. For three subjects, plasma concentrations were below the limit of quantification at 24 hours postdose. The mean plasma concentration was equal for male and female subjects for the low volumes (i.e., 50–200 μl). For the higher volumes, only one male subject was included. Interestingly, the concentration was lower than in the female subjects, especially at the peak (Supplemental Fig. 2, http://links.lww.com/MAO/C134).

On average, the meaningful plasma concentration characteristics (i.e., the area under the concentration curve zero-inf, AUC_0-inf_; the area under the curve from zero to the time of last measure above the quantification limit, AUC_0-tlast_; and the maximum concentration, *C*_max_) of AC102 were lowest in C5 (the lowest dosing cohort) and highest in C12 and C10 (the highest dosing cohorts). Plasma *C*_max_ and AUC_0-inf_ were dose proportional within the investigated dose range. For higher doses, the area under the curve (AUC_0-inf_) is higher for female subjects compared to the (single) male subject receiving the higher dose (Fig. 5B).

Plasma clearance was unchanged over the dose range, and dose proportionality was confirmed. Moreover, the highest determined *C*_max_ and AUC_0-tlast_ were well below 10% of the plasma values measured in oral toxicity animal studies (not shown) in which the NOAEL concentration was given for 28 days.

## DISCUSSION

This first-in-human, open-label, placebo-controlled, ascending dose study of a single intratympanic injection of AC102 or vehicle gel in healthy volunteers showed that the injection of AC102 in a dose up to 12 mg/ml and a maximum administered volume of 750 μl was well tolerated and safe.

Most TEAEs were assessed as mild and likely due to the administration procedure or the gel administered into the middle ear (Table 4, Supplemental Table 3, http://links.lww.com/MAO/C132). Most of the TEAEs recovered within 1 week after administration of study medication. No statistically significant association existed between the number of TEAEs in the treatment group (placebo or AC102) and the subjects' sex. Some short-term oropharyngeal pain and throat irritation were observed, likely to be related to the injection procedure, vehicle, and/or AC102 passage to the pharynx via the Eustachian tube or through activation of the auricular branch of the vagus nerve (Arnold's nerve) (22). After administration, we observed short and temporary vertigo, suggesting a caloric effect on the vestibular system. This is especially likely when injected in the posterior-inferior quadrant of the tympanic membrane (23).

Importantly, the procedure of injection did not affect the bone conduction thresholds in normal-hearing subjects, as evidenced by audiometry throughout the study, and had no long-term (i.e.,>28 d) effects on air conduction thresholds, BERA thresholds, and TEOAEs and DPOAEs. We, however, observed a temporary volume-dependent conductive hearing loss for a few days. Notably, thresholds in the higher frequencies (i.e., 4–8 kHz) showed a more significant transient conductive hearing loss than lower frequencies (i.e., 0.25–1 kHz). Analysis showed that the bone conduction thresholds were not affected. However, the frequencies of 6 and 8 kHz were not tested due to a rapid roll-off in force output and increased acoustic radiation, limiting the clinically useful range of stimulus levels to about 6 kHz in testing bone conduction thresholds (24,25).

Otitis media with effusion (OME) is the most common type of conductive hearing loss and can vary in effusion viscosity (serous vs mucoid) and volume, typically leading to a low-frequency hearing loss in the range of 18 to 35 dB or greater (26–28). At low frequencies, conductive hearing loss depends on the percentage of the original middle-ear air space that remains air filled (16) and reflects a reduction of admittance of the middle-ear air space due to the displacement of air with fluid. In contrast, at higher frequencies, hearing loss depends on the percentage of the tympanic membrane contacted by fluid. This suggests that the primary mechanism of the temporary conductive losses observed in this study is an increase in tympanic membrane mass or covering of the ossicles due to contacting gel (16). The viscosity of the thermosensitive gel seems less of a factor. Previously, it was observed that between 300 Hz and 3 kHz, there were no substantial differences in umbo velocity when fluids of different viscosities were used, and the differences were not monotonic with viscosity at any frequency (16). This study thus reveals that the transient conductive hearing loss resulting from exposure to AC102 and placebo depends on frequency, with the higher frequencies experiencing more hearing loss than the lower frequencies. This effect differs from those observed with OME, which predominantly leads to low-frequency conductive loss (26–28).

In our experience, applying a second puncture hole in the tympanic membrane enables air release from the middle ear, resulting in higher injected volumes without backflow. This was especially the case with gel volumes >200 μl. This aligns with previous work with intratympanic dexamethasone injection as salvage therapy for simultaneous bilateral SSNHL (20) or for treating refractory Menière's disease (21). More recently, an additional ventilation hole was found advantageous when applying thermosensitive hydrogels (10), the same gel used in this study. The injections were performed using two needle types (Whitacre or Quincke-type spinal needles, 25G, 90-mm length). With increasing volume, backflow was observed, and a Whitacre-type needle was used to reduce backflow and enable longer tympanic membrane visibility when no additional ventilation hole was placed (10).

One concern of injection in the middle ear, rather than the inner ear, is that the active substance does not reach the inner ear. Previous work on human cochlear pharmacokinetics (PK) measurements and modeling demonstrates that therapeutic agents can achieve efficacious levels within the cochlea, irrespective of anatomical variations such as pseudomembranes or a pronounced bony round window overhang (29). This is corroborated by Nieratschker et al., who, in a human petrous bone study, utilized the same poloxamer gel used in this report and showed sufficient coverage of the round window membrane in each injected middle ear, further challenging the prevailing notion that anatomical obstacles significantly hinder drug delivery to the inner ear (10). While anatomical variations in the middle ear, such as mucosal coverings of the round window membrane or prominent bony overhangs, are important considerations, they do not necessarily preclude the effective delivery of therapeutic agents to the inner ear.

In conclusion, the intratympanic administration of AC102 in a dose of up to 12 mg/ml and a maximum administered volume of 750 μl was well tolerated and safe. Most TEAEs were assessed as mild and likely due to the administration procedure and/or the gel administered into the middle ear. Using a temperature-sensitive gel offers the potential for sustained drug delivery to the inner ear, reducing the risk associated with direct intracochlear injection, such as inner ear inflammation and serous labyrinthitis, which could lead to long-term deafness or vestibulopathy (30). Using thermosensitive hydrogels as drug carriers, therefore, is an exciting development in inner ear therapy and represents a promising alternative to other methods, such as systemic administration and intralabyrinthine injection. A phase 2 clinical study is currently underway to assess the efficacy of AC102 for SSNHL (https://www.clinicaltrialsregister.eu/ctr-search/trial/2021-004323-33/BG).

## CONCLUSIONS

The results of this phase 1 clinical trial suggest that the intratympanic delivery of AC102 is a safe and well-tolerated approach for drug delivery to the inner ear in healthy volunteers with normal hearing. Most TEAEs were assessed as mild and likely due to the administration procedure or the gel administered into the middle ear. Injections should be performed slowly with a second air-releasing hole to achieve high volumes in the middle ear. Most of the TEAEs recovered within 1 week after administration of the study medication. Further, temporary, volume-dependent conductive hearing losses were observed in the higher frequencies. Therefore, patients should expect slight discomfort during the procedure and a short-term increase in thresholds following injection when treated for conditions such as SSNHL.
